# C7orf59/LAMTOR4 phosphorylation and structural flexibility modulate Ragulator assembly

**DOI:** 10.1002/2211-5463.12700

**Published:** 2019-07-28

**Authors:** Nadia Rasheed, Tatiani B. Lima, Gustavo F. Mercaldi, Andrey F.Z. Nascimento, Ana L.S. Silva, Germanna L. Righetto, Liron Bar‐Peled, Kuang Shen, David M. Sabatini, Fabio C. Gozzo, Ricardo Aparicio, Juliana H.C. Smetana

**Affiliations:** ^1^ Brazilian Biosciences National Laboratory (LNBio) Brazilian Center for Research in Energy and Materials (CNPEM) Campinas Brazil; ^2^ Institute of Biology University of Campinas Brazil; ^3^ Institute of Chemistry University of Campinas Brazil; ^4^ Brazilian Synchrotron Light Laboratory (LNLS) Brazilian Center for Research in Energy and Materials (CNPEM) Campinas Brazil; ^5^ The Massachusetts General Hospital Cancer Center Boston MA USA; ^6^ Department of Biology Massachusetts Institute of Technology Cambridge MA USA; ^7^ Whitehead Institute for Biomedical Research Cambridge MA USA; ^8^ Howard Hughes Medical Institute Cambridge MA USA; ^9^ Koch Institute for Integrative Cancer Research Cambridge MA USA; ^10^ Broad Institute of Harvard and Massachusetts Institute of Technology Cambridge MA USA

**Keywords:** LAMTOR, mTOR, PKA, protein phosphorylation, protein structure, Ragulator

## Abstract

Ragulator is a pentamer composed of p18, MP1, p14, C7orf59, and hepatitis B virus X‐interacting protein (HBXIP; LAMTOR 1—5) which acts as a lysosomal scaffold of the Rag GTPases in the amino acid sensitive branch of TORC1 signaling. Here, we present the crystal structure of human HBXIP‐C7orf59 dimer (LAMTOR 4/5) at 2.9 Å and identify a phosphorylation site on C7orf59 which modulates its interaction with p18. Additionally, we demonstrate the requirement of HBXIP‐C7orf59 to stabilize p18 and allow further binding of MP1‐p14. The structure of the dimer revealed an unfolded N terminus in C7orf59 (residues 1–15) which was shown to be essential for p18 binding. Full‐length p18 does not interact stably with MP1‐p14 in the absence of HBXIP‐C7orf59, but deletion of p18 residues 108–161 rescues MP1‐p14 binding. C7orf59 was phosphorylated by protein kinase A (PKA) *in vitro* and mutation of the conserved Ser67 residue to aspartate prevented phosphorylation and negatively affected the C7orf59 interaction with p18 both in cell culture and *in vitro*. C7orf59 Ser67 was phosphorylated in human embryonic kidney 293T cells. PKA activation with forskolin induced dissociation of p18 from C7orf59, which was prevented by the PKA inhibitor H‐89. Our results highlight the essential role of HBXIP‐C7orf59 dimer as a nucleator of pentameric Ragulator and support a sequential model of Ragulator assembly in which HBXIP‐C7orf59 binds and stabilizes p18 which allows subsequent binding of MP1‐p14.

**Abbreviations**
cAMP: cyclic adenosine monophosphate
Ego‐TC: Ego ternary complex
FPLC: fast performance liquid chromatography
GST: glutathione *S*‐transferase
HBXIP: hepatitis B virus X‐interacting protein
HDX: hydrogen‐deuterium exchange
HEK293: human embryonic kidney 293
MS: mass spectrometry
mTOR: mammalian/mechanistic target of rapamycin
MWCO: molecular weight cutoff
PDB: Protein Data Bank
PKA: protein kinase A
RB: Roadblock domain
SAXS: small‐angle X‐ray scattering
UPLC: ultra‐performance liquid chromatography

In order to survive in nutrient insufficient/deprived conditions, eukaryotic organisms have a signaling pathway controlled by the protein kinase mammalian/mechanistic target of rapamycin (mTOR). The mTOR is a serine/threonine protein kinase that belongs to phosphatidylinositol 3‐kinase‐related kinaseprotein family and is also known asmechanistic target of rapamycin or FK506 binding protein 12‐rapamycin associated protein 1 (FRAP1) 1, 2, 3. mTOR signaling regulates growth,proliferation, motility, protein synthesis, andtranscription. Deregulation of mTOR can be observed in cancer, type 2 diabetes, obesity, and various neurodegenerative diseases 4, 5, 6, 7.

Kim *et al*. and Sancak *et al*. 8, 9, 10, 11 independently discovered the involvement of Rag GTPases in amino acid‐dependent activation of mTORC1. In mammals, four Rag proteins form obligate heterodimers; RagA or RagB dimerizes with RagC or RagD. Amino acids promote the GTP loading on RagA/B 12, 13. The GTP bound RagA/B dimer induces the translocation of mTORC1 from cytoplasm to lysosomal surface and Rag GTPase interacts with a multisubunit complex called Ragulator 1, 14. Ragulator has emerged as an important regulator of the mTORC1 branch primarily driven by amino acids signaling. In contrast to many GTPases, Rags lack a lipid‐anchoring moiety; instead, they rely on Ragulator complex to interact with lysosomal surface.

The Ragulator complex was originally identified as a trimer composed of the MP1‐p14 dimer with p18, a lysosome‐associated adaptor protein 1, 14. Shortly after, two additional subunits were identified as an integral part of the Ragulator which was found to act as a GEF only in the presence of all five subunits 4, 14. These additional subunits are hepatitis B virus X‐interacting protein (HBXIP), an antiapoptotic protein previously known to interact with the HBx protein of the hepatitis B virus and inhibit viral replication 8, 10, 15, 16 and the uncharacterized C7orf59 protein.

The yeast counterpart of mammalian Ragulator is the Ego complex. Although the Ego complex shares functional and structural features with mammalian Ragulator such as vacuolar localization (functionally equivalent to lysosomal localization), interaction with the Rag orthologues Gtr1/Gtr2 and regulation of amino acid sensing in the conserved TOR pathway, the evolutionary and structural relationships between Ragulator and Ego subunits are less than clear. The MP1‐p14 heterodimer is related to a homodimeric Ego3 protein, while p18 shares structural features with Ego1. The putative yeast counterparts of HBXIP and C7orf59 are Ego2/Ycr075w‐a and Ego4/Ynr034w‐a 12, 17, 18. Ego2, but not Ego4, was shown to be an essential part of the Ego ternary complex (Ego‐TC) by interacting directly with Ego1 and Ego3 forming Ego‐TC which is required for the vacuolar localization of Gtr1/2 and TORC1 activation 14, 19. Ego4 interacts genetically and physically with Gtr2 but the significance of this finding is yet to be determined 14, 19.

A striking structural feature of both Ragulator/Rag and Ego/Gtr complexes is the presence of several Roadblock domains (RB), an ancient protein fold which functions as a protein interaction module. RB domains frequently interact to form either homo‐ or heterodimers that function as platforms to anchor and regulate signaling proteins such as GTPases. Structurally, the RB domain consists of α‐β‐α sandwiches of ~ 100–120 amino acid residues in which a 5‐sheet β‐meander is flanked by one α‐helix on one side (α2) and two helices on the other side (α1 and α3). Several RB proteins lack the C‐terminal α3 helix, and this variation defines a subclass of Group II RB 14, 15. Less frequently, the N‐terminal helix α1 might be missing as well, for example, in Ego2. The missing helices of these incomplete RBs can in some cases be replaced by α‐helices from their interaction partners. The MP1‐p14 heterodimer and the yeast Ego3 homodimers belong to Group I as they display all three helices characteristic of this fold, while HBXIP, C7orf59, Ego2, and Ego4 belong to Group II.

We have solved the crystal structure of the complex of human HBXIP and C7orf59 and explored its interaction with p18 and its modulation by protein kinase A (PKA). The electron density of C7orf59 displayed an unfolded, extended N terminus (residues: 1–15) which was shown to be dynamic in solution and essential for p18 binding. Truncation analysis of p18 identified different regions required for the interaction with each dimer. Our results confirm the essential role of HBXIP‐C7orf59 dimer as an integral part of pentameric Ragulator and support a sequential model of Ragulator assembly in which HBXIP‐C7orf59 stabilizes p18 and allows subsequent binding of MP1‐p14. These findings are in agreement with the recently reported structural studies of pentameric Ragulator 20, 21, 22, 23, 24.

## Results

### Structure of the HBXIP‐C7orf59 dimer reveals an unstructured N terminus on C7orf59 which is essential for p18 binding

**Figure 1:**
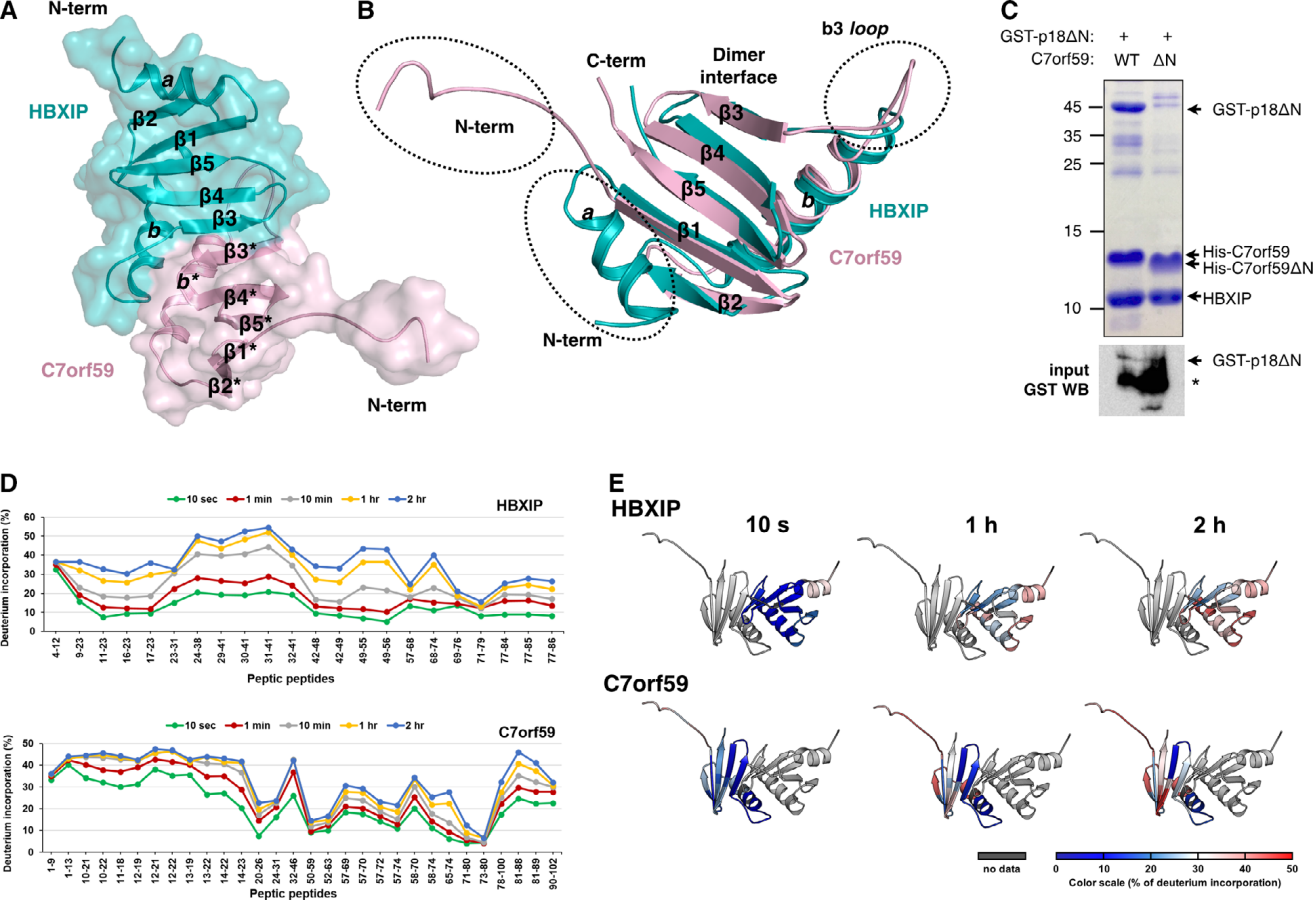
Structure and dynamics of the HBXIP‐C7orf59 dimer. (A) Cartoon/space filling representation of the structure of HBXIP‐C7orf59 dimer. The structural elements are named according to Kurzbauer *et al*., 15, and asterisks indicate structural elements or residues from C7orf59. (B) Superposition of C7orf59 (pink) and HBXIP (cyan) highlighting the differences in conformation of loop *b*3 and the N terminus. (C) Talon pulldown assay of GST‐p18ΔN with HBXIP‐C7orf59 dimer containing either His‐C7orf59 WT or ΔN mutant (residues: 16–99) shows that the N terminus of C7orf59 is essential for its interaction with p18. Upper panel: Coomassie‐stained SDS/PAGE of the pulldown (bound fraction). Lower panel: GST western blot of the lysates (input) showing comparable amounts of GST‐p18ΔN. The asterisk indicates a degradation product of GST‐p18ΔN. (D) HDX plot displaying the percentage of deuterium incorporation for each of the peptic peptides identified in HBXIP (upper) and C7orf59 (lower). (E) Structural representation of the relative deuterium incorporation of C7orf59 and HBXIP. Relative deuterium exchange (%) is represented from blue to red as indicated in the scale below.

We expressed and purified the HBXIP‐C7orf59 dimer in *Escherichia coli* from a bicistronic plasmid, recovering stoichiometric amounts of both proteins. The HBXIP‐C7orf59 dimer crystallized in space group C2 with four dimers in the asymmetric unit (Table 1). The structure was deposited under Protein Data Bank (PDB) i.d. 5VOK. The crystals were small, plate‐like and diffracted up to 2.95 Å. The overall structure and dimer interface of HBXIP‐C7orf59 are similar to previously reported structures 21, 23 except for the presence of defined electron density corresponding to the unfolded N‐terminal region (residues: 1–15) in C7orf59 (Figs 1A,B and S1). Comparison with the pentameric Ragulator complex 20, 21, 22, 23, 24 shows that the N terminus of C7orf59 undergoes a transition to alpha‐helical conformation upon binding of p18. Strikingly, deletion of residues 1–15 of C7orf59 severely impairs the dimer's interaction with p18, highlighting its importance in stabilizing the complex (Figs 1C and S2D).

**Table 1:** Data collection and refinement statistics. Statistics for the highest resolution shell are shown in parentheses.

	HBXIP‐C7orf56
Wavelength (Å)	0.9686
Resolution range (Å)	81.85–2.89 (3.07–2.89)
Space group	C 1 2 1
Cell dimensions	
*a*, *b*, *c* (Å)	180.88, 64.76, 79.23
α, β, γ (°)	90, 115.18, 90
Total reflections	125 792 (20 673)
Unique reflections	18 719 (3004)
Multiplicity	6.7 (6.9)
Completeness (%)	99.5 (99.1)
Mean *I*/sigma(*I*)	6.5 (0.7)
Wilson *B*‐factor (Å^2^)	86.24
*R*‐merge (%)	17.1 (350)
*R*‐means (%)	18.6 (379)
CC1/2 (%)	99.3 (33.7)
*R*‐work (%)	28.0
*R*‐free (%)	31.2
Number of nonhydrogen atoms	5252
Macromolecules	5250
Solvent	2
Protein residues	705
RMS deviation (bonds; Å)	0.008
RMS deviation (angles; °)	1.40
Ramachandran favored (%)	91.53
Ramachandran allowed (%)	7.88
Ramachandran outliers (%)	0.58
Rotamer outliers (%)	1.86
Clashscore	34.54
Average* B*‐factor (Å^2^)	128.41
Macromolecules	128.43
Solvent	89.14
Number of TLS groups	46
PDB code	5VOK

The purified HBXIP‐C7orf59 dimer was analyzed by hydrogen/deuterium exchange‐mass spectrometry (HDX‐MS) to evaluate its conformational dynamics and support crystallographic data (Figs 1D,E and S3). In the C7orf59 structure, the regions showing highest relative deuterium incorporation were the unfolded N terminus, sheets β2 and β5 and the N‐terminal side of helix b*. These regions displayed up to 50% deuterium incorporation after 2 h. For HBXIP, the overall deuterium incorporation was lower, indicating a more rigid fold. The only region where incorporation rates reached 50% in HBXIP was the C‐terminal side of helix *b*, which is adjacent to the N terminus of the corresponding helix in C7orf59 which also displayed high deuterium incorporation. The *b*3* loop displayed low deuterium incorporation, consistent with its tight interaction with the 2*b* pocket. These results confirm the unfolded nature of the C7orf59 N terminus and highlight additional regions with high conformational flexibility.

### Interaction studies suggest that pentameric Ragulator is assembled sequentially from HBXIP‐C7orf59‐p18 trimer

**Figure 2:**
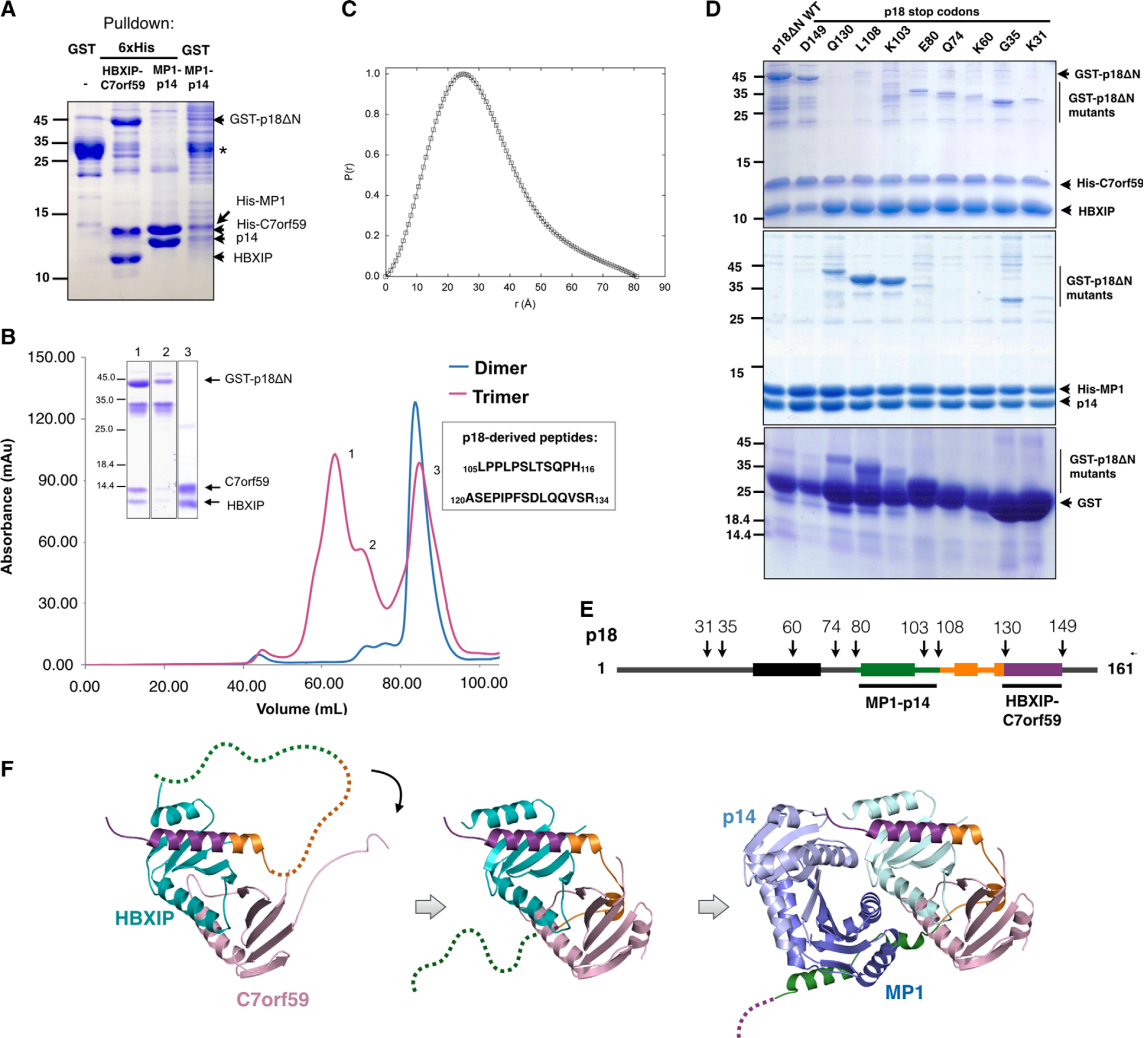
Sequential assembly of the Ragulator complex. (A) Affinity‐purified GST‐p18ΔN migrates as a ~ 35 kD band on SDS/PAGE (indicated with an asterisk), while its complex with HBXIP‐C7orf59 results in a band of ~ 44 kD indicating that GST‐p18ΔN is protected from degradation in the presence of HBXIP‐C7orf59. The sample shown in the first lane was purified using Glutathione‐Sepharose beads (GST pulldown) and the second and third using Talon beads (6xHis pulldown). The fourth lane is similar to the third lane, except that pulldown was performed using Glutathione‐Sepharose beads instead of Talon. (B) Size exclusion elution profiles of the HBXIP‐C7orf59 dimer (blue) and the dissociation products of the GST‐p18ΔN + HBXIP‐C7orf59 complex (magenta) on a Superdex 200 16/60 column, indicating the sequences of p18‐derived peptides identified in peak 3. Inset: Coomassie‐stained SDS/PAGE analysis of representative fractions from the three peaks. (C) Pair–distance distribution function of HBXIP‐C7orf59 dimer bound to p18‐derived fragments (peak 3 in panel B). The dimer used in SAXS analysis was his‐tagged. (D) Mapping of p18 binding to HBXIP‐C7orf59 and MP1‐p14. Stop codons were inserted in GST‐p18ΔN sequence in the indicated positions by site‐directed mutagenesis, and the resulting constructs were coexpressed either with HBXIP‐C7orf59 (upper panel) or MP1‐p14 (middle panel) followed by Talon pulldown. The GST‐p18ΔN stop codon constructs were expressed and isolated by GST pulldown to analyze the effects of C‐terminal truncations on p18 degradation (lower panel). (E) Schematic representation of the p18 secondary structure and the constructs used in the stop codon scanning (arrows). The numbers indicate the positions of stop codon insertion by site‐directed mutagenesis, and rectangles indicate the positions of α‐helices in p18 structure. (F) Schematic model of sequential Ragulator assembly. The images were prepared using PDB: 5VOK (first image) and 6EHP (second and third images). p18 is colored according to the regions defined in (E).

The HBXIP‐C7orf59 dimer was coexpressed in *E. coli* with an N‐terminally truncated p18 construct (p18ΔN, residues: 6–161) in fusion with an N‐terminal glutathione *S*‐transferase (GST) tag. When GST‐p18ΔN was expressed alone in *E. coli* and purified from the soluble fraction by GST pulldown, a major band of 35 kD was detected indicating proteolytic degradation. Coexpression of the same GST‐p18ΔN construct with HBXIP‐C7orf59 followed by Talon pulldown yielded a 44 kD band, compatible with the predicted molecular weight of the GST‐p18ΔN fusion protein, showing that recombinant HBXIP‐C7orf59 dimer interacts with GST‐p18ΔN and prevents its degradation. Under the same conditions, coexpression with MP1‐p14 failed to promote stabilization and recovery of full‐length GST‐p18ΔN (Fig. 2A).

Size exclusion chromatography of the affinity‐purified HBXIP‐C7orf59/GST‐p18ΔN trimeric complex resulted in three peaks originating from spontaneous degradation of GST‐p18ΔN and dissociation of HBXIP‐C7orf59 dimer from the complex. SDS/PAGE and western blot analysis showed that the first peak corresponded mostly to intact HBXIP‐C7orf59/GST‐p18ΔN complex, although contaminated with a p18 degradation product, followed by a second peak characterized by further degradation of p18 and full dissociation of HBXIP‐C7orf59, and a third peak of HBXIP‐C7orf59 dimer alone (Figs 2B and S4). The apparent molecular weight of the p18 degradation intermediate in SDS/PAGE analysis (~ 35 kD) indicates that it consists of GST in fusion with the N‐terminal half of p18, suggesting that it dissociates from HBXIP‐C7orf59 upon loss of its C‐terminal half. LC/MS analysis on Synapt G1 HDMS after trypsinization of samples from peak 3 of Fig. 2B detected nontryptic peptides _105_LPPLPSLTSQPH_116_ and _120_ASEPIPFSDLQQVSR_134_, derived from the C‐terminal half of p18 sequence, confirming that peak 3 contains p18 proteolytic fragments bound to HBXIP‐C7orf59. Both HBXIP and C7orf59 were identified in peak 3 with high sequence coverage, thus validating the analysis (not shown).

Representative fractions from the first and third peaks of size exclusion were analyzed by small‐angle X‐ray scattering (SAXS). The first peak showed signs of aggregation which prevented further analysis. For the third peak, the linear Guinier region of the scattering curve indicated that this sample was monodisperse. SAXS parameters (Rg: 2.29 nm, *D*_max_: 7.83 nm, MW: 29.1 kDa) supported the idea that this peak corresponds mostly to HBXIP‐C7orf59 dimer associated with p18‐derived peptides (Fig. 2C). An *ab initio* model built from this curve indicates that these peptides bound to HBXIP‐C7orf59 in peak 3 do not contribute significantly to increase the volume of the dimer, and might in fact promote a more compact shape (Fig. S5).

Stop codons were inserted by site‐directed mutagenesis along the sequence of GST‐p18ΔN to map the interactions with HBXIP‐C7orf59 and MP1‐p14 dimers. These mutants were expressed in *E. coli*, and Talon pulldowns were performed with coexpressed HBXIP‐C7orf59 or MP1‐p14. GST pulldowns of each construct expressed alone were performed to verify the effects of these mutations on the expression levels and stability of GST‐ p18ΔN. Both full‐length and 149_stop mutant of p18 interacted strongly with HBXIP‐C7orf59 dimer, while all the other mutations substantially decreased this interaction (Fig. 2D). Surprisingly, deletion of residues 130–161 of p18 strongly favored the interaction with MP1‐p14, which was essentially undetectable both for full‐length p18 and 149_stop mutant (Fig. 2D). This interaction was enhanced even further by deletion of residues 103–161 and 108–161 of p18, which correlates with the intrinsic stabilizing potential of these truncations, observed when GST‐p18ΔN and its deletion mutants were expressed alone (Fig. 2D, lower panel).

*In vitro* assembly of the pentamer from affinity‐purified MP1‐p14 dimer and HBXIP‐C7orf59/GST‐p18ΔN trimer confirmed that recombinant GST‐p18ΔN is capable of binding to MP1‐p14 after previously assembled in a trimer with HBXIP‐C7orf59. The results of size exclusion chromatography indicated the formation of a pentamer which showed a dissociation/degradation pattern comparable to that of HBXIP‐C7orf59/GST‐p18ΔN trimer (Fig. S6). Taken together, these results indicate that the preferred pathway to Ragulator assembly starts from HBXIP‐C7orf59‐p18 trimer with further incorporation of MP1‐p14. Interaction with HBXIP‐C7orf59 prevents degradation of p18 and probably stabilizes it in the right conformation to bind MP1‐p14. The truncated mutants of p18 at positions Q130, L108, and K103 bind strongly to MP1‐p14 in the absence of HBXIP‐C7orf59, probably because the truncations eliminate an inhibitory region of p18 while retaining the MP1‐p14 binding site (Fig. 2E,F).

### PKA phosphorylates C7orf59 on Ser67 and negatively regulates Ragulator assembly

**Figure 3:**
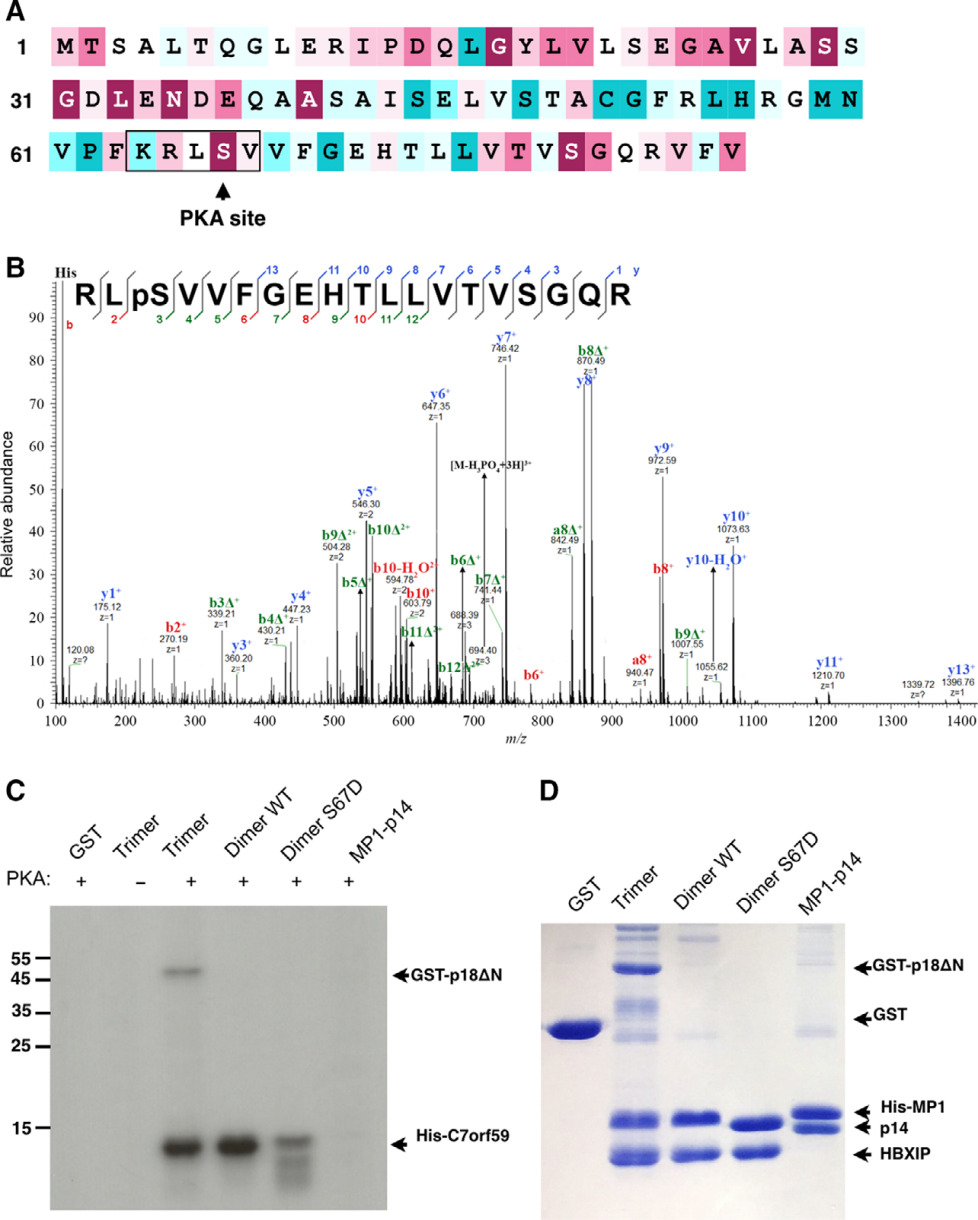
PKA phosphorylates C7orf59 on Ser67. (A) Sequence of C7orf59 was color‐coded from less (cyan) to more conserved (burgundy) using the Consurf server (consurf.tau.ac.il) 58, 59, 60 and the potential PKA site was highlighted. (B) MS/MS fragmentation spectrum of a phosphorylated peptide of immunoprecipitated FLAG‐C7orf59 from unstimulated HEK293 cells, confirming the phosphorylation of C7orf59 on Ser67. (C) Autoradiography of *in vitro* phosphorylation of purified Ragulator components with PKA showing the phosphorylation of C7orf59, which is prevented by mutation of Ser67. (D) Coomassie‐stained SDS/PAGE analysis of the protein substrates used in the kinase assay.

Sequence analysis with kinasephos 2.0 9, 25, 26 identified Ser67, an evolutionarily conserved residue, as a potential PKA phosphorylation site with high score (Fig. 3A). This phosphorylation was detected by MS analysis in immunoprecipitated FLAG‐C7orf59 from human embryonic kidney 293T (HEK293T) cells (Fig. 3B). To confirm that this site is phosphorylated by PKA, purified samples of HBXIP‐C7orf59 dimer (WT and S67D), HBXIP‐C7orf59/GST‐p18ΔN trimer, MP1‐p14 dimer, and GST were phosphorylated *in vitro* with PKA. Incorporation of radiolabeled phosphate demonstrated that C7orf59 is strongly phosphorylated by PKA, and mutation of Ser67 to aspartate reduced the extent of phosphorylation, indicating that this is the major PKA phosphorylation site on C7orf59. GST and other Ragulator subunits were not phosphorylated under the same conditions, except for p18, which was weakly phosphorylated. Comparing the HBXIP‐C7orf59 dimer, with HBXIP‐C7orf59/GST‐p18ΔN trimer, the presence of p18 slightly reduced the intensity of C7orf59 phosphorylation (Fig. 3C,D).

**Figure 4:**
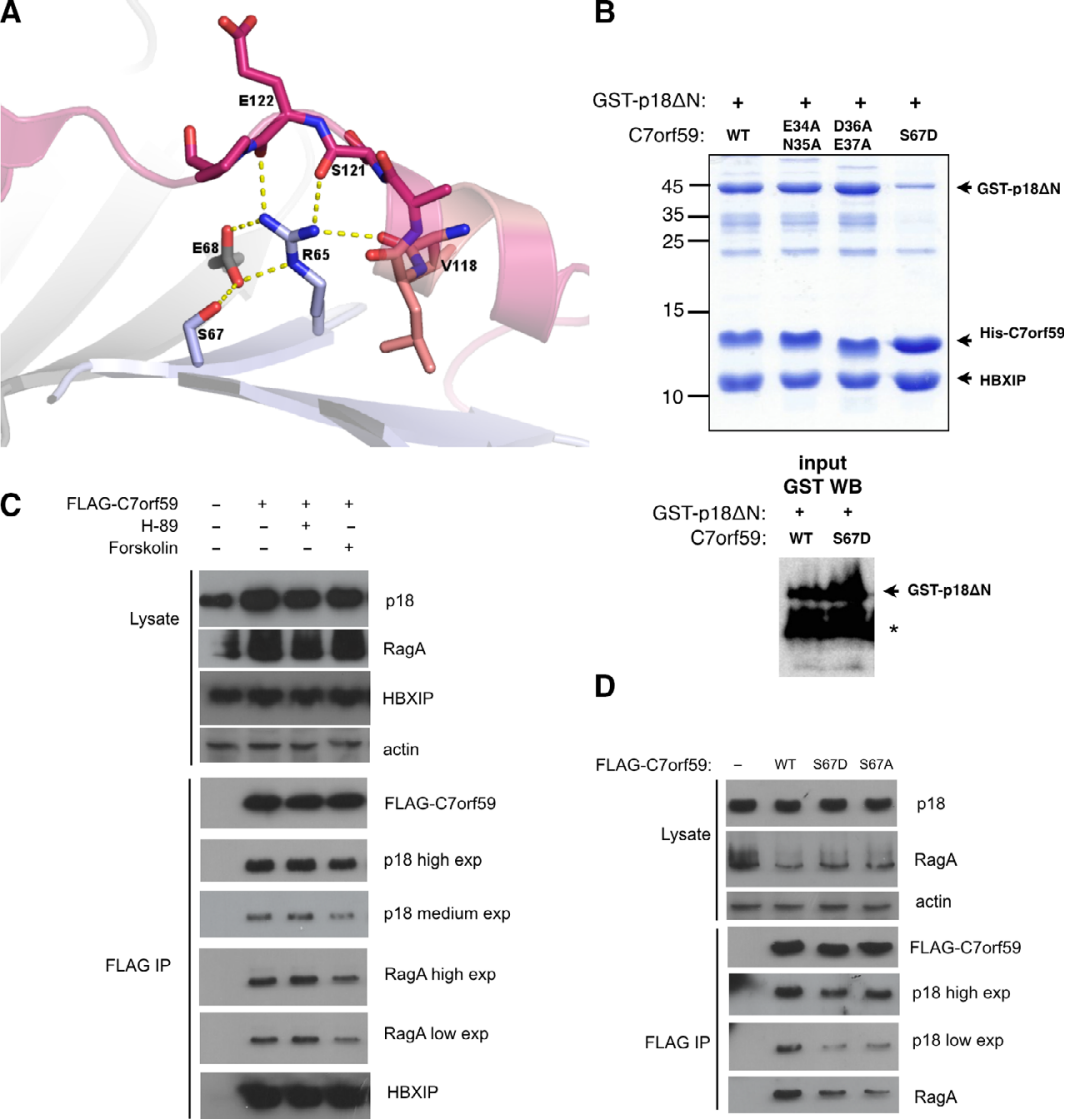
Stability of the Ragulator complex is affected by PKA phosphorylation of C7orf59. (A) Ser67 is involved in a network of polar contacts with residues from p18 (Val118, Ser121, Glu122), HBXIP (Glu68), and C7orf59 (Arg65). The image illustrates the interface of p18 (pink), HBXIP (light gray), and C7orf59 (light blue) from PDB: 5Y39 21. (B) Mutation of Ser67 to aspartate impairs p18 binding. Left panel: The Talon pulldown assay of GST‐p18ΔN with each C7orf59 mutant was analyzed by Coomassie‐stained SDS/PAGE. Only S67D mutant shows substantial decrease in the interaction with GST‐p18ΔN. Right panel: GST western blot of the lysates (input) showing comparable amounts of GST‐p18ΔN coexpressed with C7orf59 WT and S67D. The asterisk indicates a degradation product of GST‐p18ΔN. (C, D) Effect of PKA modulation and Ser67 mutations on the Ragulator complex. HEK293‐T cells were transiently transfected with FLAG‐C7orf59 or mutants and treated with PKA modulators (Forskolin and H‐89) as indicated. The interactions of FLAG‐C7orf59 with endogenous proteins were detected by immunoprecipitation using FLAG‐specific antibody and western blot using antibodies specific to FLAG epitope, p18, HBXIP, and RagA.

C7orf59 Ser67 is located in strand β3 and is involved in polar contacts with HBXIP Glu68 and C7orf59 Arg65 in the pentamer structure. The structural context of this residue indicates that its phosphorylation would interfere in a network of polar contacts with p18 (Fig. 4A). To investigate this possibility, Ser67 was mutated to aspartate to mimic the charge effects of phosphorylation, which severely diminished the dimer's association with p18 in an *in vitro* pulldown assay using recombinant proteins. In comparison, mutation of Glu34/Asn35 or Asp36/Glu37, which are also highly conserved, did not affect the dimer integrity or p18 binding. (Figs 4B and S2).

To investigate the consequences of C7orf59 phosphorylation by PKA in the cell, we expressed C7orf59 in HEK293‐T cells as an N‐terminal FLAG fusion and the transiently transfected cells were stimulated with forskolin, an activator of adenylate cyclase which is widely used as a PKA agonist 27. The C7orf59 interactions with p18 and RagA were reduced in the presence of forskolin, indicating that PKA activation results in breakdown of the Ragulator complex. To further confirm that this effect could be attributed to PKA activation, we used the PKA inhibitor H‐89. Preincubation with this inhibitor prevented the dissociation induced by forskolin, supporting the specific involvement of PKA (Figs 4C and S2). In agreement with the hypothesis that PKA activation by forskolin induces phosphorylation of C7orf59 on Ser67, thereby impairing its interaction with p18, we observed that mutation of this residue to aspartate mimics the effect of PKA activation on C7orf59 interactions with p18 and RagA. Mutation of the same residue to alanine also impaired the interactions, suggesting that the polarity of the serine residue is essential for the stability of the complex (Figs 4D and S2).

## Discussion

Our study highlights the role of C7orf59 as an important component of the Ragulator, acting as a nucleating factor for p18 and as a phosphorylation target. Comparison of the apo HBXIP‐C7orf59 structure solved in this study with the HBXIP‐C7orf59 dimer present in the pentameric Ragulator 20, 21, 22, 23, 24 demonstrates that the unfolded N terminus of C7orf59 (residues: 1–15) undergoes a structural rearrangement upon p18 binding, adopting a helical structure similar to the N terminus of other Roadblock subunits. Although the lack of electron density in the N‐terminal region of C7orf59 in other structures of unbound C7orf59‐HBXIP dimer 21, 23 and HDX analysis 20 suggested that this region is unfolded, our structure is the first to capture the full N terminus of C7orf59 in an extended conformation due to its unique crystal packing. The strict requirement for the N terminus of C7orf59 for binding to p18, demonstrated by deletion of this region, highlights the importance of its flexible nature. Interestingly, C7orf59 is the only Roadblock subunit in the Rag‐Ragulator complex which undergoes significant conformational changes.

The p18 regions assigned here as responsible for stable binding to HBXIP‐C7orf59 (residues: 130–149) and MP1‐p14 (residues: 80–108) by truncation and pulldown analysis are found associated with HBXIP and MP1, respectively, in the structure of the pentamer 20, 21, 22, 23, 24. The two p18‐derived peptides which copurified with HBXIP‐C7orf59 dimer obtained from dissociation of the trimer are part of the interface with C7orf59 in the pentamer structure. The proline‐rich peptide _105_LPPLPSLTSQPH_116_ corresponds to an extended region of p18 which is tightly packed in the hydrophobic *b*3 pocket of HBXIP. Peptide _120_ASEPIPFSDLQQVSR_134_ corresponds to the N‐terminal side of the long C‐terminal helix of p18 which binds to the β‐sheet surface of HBXIP, and the missing region between these peptides (residues: 117–119) corresponds to the center of the short helix that binds to the exposed beta‐sheet of C7orf59. Importantly, we were able to assemble pentameric Ragulator from separated HBXIP‐C7orf59‐p18 trimer and MP1‐p14 dimer but we did not observe MP1‐p14 binding to p18 in the absence of HBXIP‐C7orf59. MP1‐p14 dimer fails to bind and stabilize full‐length p18, while truncated mutants of p18 display enhanced binding to this dimer. These results support a hierarchical model of Ragulator assembly in which HBXIP‐C7orf59 is responsible for the initial nucleation of the complex, stabilizing p18 in a conformation which allows subsequent binding of MP1‐p14. This insight is complimentary to the publications of high‐resolution structures of the assembled pentamer and agrees with the observation by Yonehara *et al*. 22 of the existence of a ‘pre‐Ragulator’ formed by p18, C7orf59, and HBXIP.

Additionally, our study identified a new phosphorylation site on C7orf59 which impacts on Ragulator assembly. We confirmed phosphorylation of Ser67 by MS analysis of C7orf59 from transfected cells and *in vitro* phosphorylation of purified Ragulator subunits with PKA. In transfected cells, the C7orf59‐p18 interaction was negatively affected by activation of cyclic adenosine monophosphate (cAMP)‐dependent PKA and by mutation of Ser67 to aspartate or alanine. These results suggest that PKA phosphorylation of C7orf59 Ser67 induces the regulated breakdown of the Ragulator complex. This observation agrees with our biochemical studies which indicated that targeting the c7orf59 interface with p18 leads to breakdown of the whole pentamer.

Protein kinase A is involved in metabolic regulation downstream of G‐protein‐coupled receptor/cAMP signaling, and one of its important upstream activators is the hormone glucagon 28. Therefore, PKA is activated in response to nutritional scarcity to stimulate catabolism, in contrast with mTOR, which is activated by nutrient availability to stimulate anabolism. It would not be surprising if these two pathways could negatively regulate each other. However, while it is widely accepted that there exists a PKA‐mTORC1 crosstalk, the exact mechanisms are not completely understood and cAMP may either activate or inactivate mTOR depending on cell type 2, 3, 29. Our results suggest a mechanism for mTORC1 regulation by PKA involving direct modulation of Ragulator stability by PKA activation. Further investigation of this novel mechanism might shed light on the crosstalk of these important pathways.

## Methods

### Plasmid construction and mutagenesis

The pET‐Duet‐C7orf59‐HBXIP bicistronic expression plasmid was obtained by subcloning both cDNAs sequentially into a modified version of pET‐Duet‐1 plasmid (Clontech, Mountain View, CA, USA) harboring a PreScission site. C7orf59 was cloned between the *BamHI *and *NotI *sites in frame with an N‐terminal hexahistidine tag followed by the PreScission protease cleavage site, and HBXIP was cloned between *NdeI *and *XhoI *restriction sites. The pACYC‐Duet‐C7orf59‐HBXIP plasmid was constructed by subcloning the bicistronic cassette 6xHis‐PreScission‐HBXIP‐C7orf59 from pET‐Duet‐C7orf59‐HBXIP into pACYC‐Duet (Clontech) using the *NcoI *and *XhoI *restriction sites. p18ΔN was subcloned into pGEX‐4T‐1 from pET302. C7orf59 was subcloned from pACYC‐Duet into pCDNA‐FLAG (*BamHI *and *NotI*). MP1‐p14 expression plasmid was a kind gift from T. Clausen 15. Site‐directed mutagenesis was performed by thermal cycling with *Pfu *(Thermo Scientific, Waltham, MA, USA) and digestion of parental DNA with *DpnI *(Thermo Scientific), followed by transformation into DH5αand clone confirmation by sequencing. A total of nine stop codon mutants of GST‐p18ΔN were constructed (D149, Q130, L108, K103, E80, Q74, K60, G35, K31). Three mutants of C7orf59, that is, E34A/N35A, D36A/E37A, and S67D, were also designed to target the conserved residues of the protein. pACYC‐C7orf59ΔN‐HBXIP was obtained using Phusion polymerase. All the mutant clones were confirmed through sequencing. The sequences of mutagenic primers are given in Table S1.

### Affinity purification

Expression of His‐C7orf59‐HBXIP was induced with 0.5 mm IPTG at 30 °C for 16 h at 200 r.p.m. in 20 L of LB medium. Large‐scale affinity purification of 6XHis‐C7orf59‐HBXIP was performed using PBS pH 7.4 (1X) with 5% glycerol, 2 mm β‐mercaptoethanol, and 5 mm of imidazole as resuspension/lysis buffer. The resuspended lysate was supplemented with 1 mm PMSF and protease inhibitor cocktail (1X), incubated with lysozyme (0.1 mg·mL^−1^) for 1 h at 4 °C on ice and then lysed by sonication (5–6 cycles of 15 s at 1‐min interval apart). The cleared lysate obtained after centrifugationat 16 000 ***g*** for 1–2 h at 4 °Cwas subjected to batch affinity purification using Talonresin (Clontech). After incubation with resin, lysate was centrifuged at 650 r.p.m. at 4 °C to remove the flow through. The Talon beads were washed five times with the resuspension buffer, and proteins were eluted withPBS pH 7.4 (1X) supplemented with 300 mm imidazole, 5% glycerol, and 2 mm β‐mercaptoethanol. The cleared lysates were divided into two batches before proceeding to the washing and elution steps. To remove the excess imidazole, the sample was dialyzed in PBS buffer (1X) pH 7.4 with 5% glycerol, 2 mm β‐mercaptoethanoland later cleaved with 80 units of PreScission protease (GE Healthcare, Chicago, IL, USA) for 16 h at 4 °C to remove the His tag from C7orf59.

### Size exclusion chromatography and crystallization of HBXIP‐C7orf59

The cleaved sample was concentrated to 2 mL using a centrifugal filter device [GE Healthcare, molecular weight cutoff (MWCO) 3.000/3kDa], and size exclusion chromatography was performed on a Superdex 200 column 16/60 (GE Healthcare) connected to an ÄKTA fast performance liquid chromatography (FPLC) system using 50 mm Tris/HCl (pH 7.4) supplemented with 2 mm of β‐mercaptoethanol, 5% glycerol, and 100 mm of NaCl. UV absorbance was monitored at 280 nm to indicate the presence of proteins. The peak fractions were collectedandconcentrated using a centrifugal filter device (GE Healthcare, MWCO 3.000/3 kDa) until the desired concentration was achieved for the follow‐up crystallization experiments. The final concentration of the pure protein was determined from the UV absorption at 280 nm using a NanoDrop spectrophotometer. ProtParam was used for the calculation of molar extinction coefficient of proteins (http://www.expasy.ch/tools/protparam.html). The initial crystallization attempts were performed in the high‐throughput crystallization facility of the Brazilian National Biosciences Laboratory, RoboLab. Crystals were grown by vapor diffusion (hanging drop) at 18 °C. Diffraction quality crystals were fully grown after 25–30 days in conditions: 4 m sodium formate with 5% glycerol (as a main refinement condition) and 0.01 m barium chloride as an additive.

### Data collection and structure solution

Native data for HBXIP‐C7orf56 crystals were collected at 100 K on the I24 beamline at the Diamond Light Source (Didcot, UK). Data sets were processed by the automatic pipeline at beamline with xia2 using dials 30, 31 and then scaled and merged with aimless using CC_1/2_cutoffs 32, 33, 34. Significant anisotropy of crystallographic data was detected at this stage, probably due to crystal plate‐like morphology. Initially, the data sets have been automatically assigned to one of the space groups I222, C2, or P1. The phase problem was firstly solved using the best crystal, indexed in space group I222. An automated molecular replacement procedure as implemented in mrbump 34, 35 was employed, using as search models PDB files for HBXIP (3MSH, 3MS6) and an *in silico *modeling for C7orf59 built on the Phyre2 server (http://www.sbg.bio.ic.ac.uk/phyre2) 26, both in their original forms and as additional modified models obtained with pdbclip 34, 36 chainsaw 37, sculptor 38 *,* and pdbset 34. Multiple alignment and structural analysis were done with clustalw2 39 and scop 40, respectively. After automated model preparation, molecular replacement solutions were obtained independently with molrep 36 and phaser 41 and subjected to a preliminary refinement with refmac5 42.

A total of 23 solutions have been output by mrbump, the top solution with a *R*_free_value of 0.493. Curiously, overall inspection with pymol 43 showed that, with a few exceptions, most of the solutions corresponded to a same orientation within the asymmetric unit (with crystallographic symmetry taken into account if necessary). Surprisingly, a representative of the main solution cluster, the top solution, which was found by phaser, was complementary to a different orientation found by molrep, as judged by the expected HBXIP‐C7orf59 dimer interface obtained by* in silico* modeling. These two partial solutions were then manually combined to give an initial model. After a few rounds of refinement and model rebuilding, *R*_free _decreased to 0.425. Despite the indication of a successful solution, it was difficult to obtain a reliable and stable refinement, which led us to consider a lower symmetry space group. Further refinement then proceeded to completion with data from the same crystal now processed in space group C2, using the available pre‐refined model in a simple molecular replacement procedure done with phaser. All through the process, Free r flag was the same, ensuring the accuracy of the crystal structure. The model was improved by iteratively model rebuilding in coot 44 and refined using refmac5, pdb_redo, and phenix.refine 34, 42, 45, 46, 47. In the last refinement stages, twin refinement was performed with phenix.refine using the twin law (‐h‐2l, ‐k, l; fraction of 0.32) identified by phenix.xtriage 48. Data collection and refinement statistics are given in Table 1. pymol 1.7 was used to prepare the crystallographic models figures.

### Pulldown assays

The expression plasmids for GST‐p18ΔN, HBXIP‐C7orf59, or MP1‐p14, or the respective mutated constructs were cotransformed into *E. coli *BL21 (DE3) and expression was induced by adding 0.5 mm IPTG and incubating for 16 h at 30 °C at 200 r.p.m. The pellets were lysed using 0.1 mg·mL^−1^ Lysozyme combined with sonication in pH 7.4PBS buffer supplemented with 5% glycerol, 2 mm β‐mercaptoethanol, and 5 mm of imidazole (the latter used only for Talon pulldowns). Glutathione‐Sepharose beads (Glutathione‐Sepharose 4B; GE Healthcare) or Talon beads (Clontech) were added to the cleared lysates, which were incubated for 3 h at 4 °C on an agitator at a very slow speed. The samples were washed five times with lysis buffer, and the beads were eluted withLaemmli buffer and analyzed by Coomassie‐stained SDS/PAGE 15%. We chose Talon beads over Glutathione resin (GST beads) to carry out the pulldown experiments involving dimers with p18 constructs to promote enrichment of p18, which is associated with the dimer complex, by capturing his‐tagged C7orf59 or MP1 instead of GST‐tagged p18.

### Assembly of pentameric Ragulator

For the assembly of pentameric Ragulator complex, both HBXIP‐C7orf59/GST‐p18ΔN trimer and MP1‐p14 dimer complexes were purified using the same purification protocol as HBXIP‐C7orf59 dimer except His‐tags were not cleaved. The complexes were mixed, and concentrated sample was passed through Superdex 200 column 10/30 (GE) connected to an ÄKTA FPLC system using 50 mm Tris/HCl (pH 7.4) supplemented with 2 mm β‐mercaptoethanol, 5% glycerol, and 100 mm NaCl.

### Hydrogen‐deuterium exchange and data processing

Hydrogen‐deuterium exchange was started by diluting the sample of purified HBXIP‐C7orf59 1 : 15 in deuterated buffer (50 mm Tris/DCl, 100 mm NaCl, 2 mm β‐mercaptoethanol, 5% glycerol, pD 7.4) at 25 °C and stopped at different time points (10 s, 1 min, 10 min, 1 h and 2 h) by adding equal volumes of quench buffer [800 mm Guanidine. HCl, 0.8% formic acid (v/v), 20 mm DTT, pH 2.5] at 4 °C. A control sample was collected at time = 0. Samples were injected into a nanoAcquity ultra‐performance liquid chromatography (UPLC) system with HDX technology coupled to Synapt G1 HDMS (Waters Corporation, Milford, MA, USA). Mobile phase A was 0.1% (v/v) formic acid in water. Mobile phase B was 0.1% (v/v) formic acid in acetonitrile. Online digestion was performed on an immobilized pepsin column (2 × 30 mm; Applied Biosystems, Foster City, CA, USA) for 4 min at 15 °C with 35 µL·min^−1^ flow. The resulting peptides were desalted on an ACQUITY UPLC BEH C18 pre‐column (1.7 µm, VanGuard, Waters Corporation) at 0 °C and separated running a linear gradient from 10% to 40% mobile phase B on an analytical column (ACQUITY UPLC BEH C18 1.7 µm, 1 mm × 100 mm; Waters) at 0ºC with 50 µL·min^−1^ flow over 12 min. MS analysis of eluting peptides was performed by data‐independent acquisition (HDMS^E^). The peptides were introduced into the mass spectrometer via LockSpray dual electrospray ion source (Waters Corporation) and a capillary voltage of 3.5 kV. The source and desolvation temperatures were set at 80 and 175 °C, respectively. Precursor ion information was collected in low‐energy MS mode at constant collision energy of 5 V. Fragmentation ion information was obtained in elevated energy scan applying 30 V. MS data were acquired in the range 50–1700 *m/z*. Phosphoric acid was used as lock mass at 0.05% (v/v) in water: acetonitrile (1 : 1, v/v) and sampled every 23 s into the mass spectrometer via the reference sprayer of the LockSpray source. The runs were processed by Protein Lynx Global Server v.2.4 (Waters Corporation) and dynamx v.3.0 (Waters Corporation). For C7orf59, a total 33 of peptides were detected, with 96.1% sequence coverage, and 3.76 redundancy, while for HBXIP, a total 34 of peptides were detected, with 91.3% coverage and 4.00 redundancy (Fig S3).

### Small‐angle X‐ray scattering

Small‐angle X‐ray scattering curves were recorded at the SAXS‐1 beamline at Brazilian Synchrotron Light Laboratory, LNLS, equipped with a Dectris Pilatus 300K detector (84 mm × 107 mm) and a capillary sample holder. Sample‐to‐detectordistancewas 1606.50 mm, and radiation wavelength was 1.55 Å, with *q *ranging from 0.0063 to 0.2787 Å^−1 ^(*q* = 4πsinθ/λ, where 2θ is the scattering angle). Fractions from size exclusion chromatography were collected and analyzed immediately after centrifugation at 13 000 ***g*** for 10 min at 4 °C, and the size exclusion buffer was used as reference for buffer subtraction. A series of increasing exposure times (5, 30, 90 s) was employed to assess potential radiation damage. Fit2D 49 was used for data integration, normalization to the intensity of the transmitted beam and sample attenuation, and buffer scattering subtraction. Absolute calibration of scattering data was performed using water as a secondary standard 50, 51. atsas 52 and gnuplot (http://www.gnuplot.info) were used for data analysis and plotting. The radius of gyration (Rg) and the zero‐angle scattering intensity (*I*(0)) were obtained from the Guinier approximation *I*(*q*) = *I*(0)exp(−*q*2Rg2/3), valid for *q*Rg ≲ 1.3, and by the indirect Fourier transform method implemented in the program GNOM 53. The pair‐distance distribution function, *P*(*r*), was calculated from the scattering curve using GNOM. Molecular mass was estimated by a concentration‐independent method based on the Porod invariant (∫*q* ^2^ *I*(*q*)*dq*). For model construction, forty dummy atom models of the HBXIP‐C7orf59 dimer were generated from the scattering curve using DAMMIF 54 in mode ‘slow’, without enforcing any symmetry (P1) or anisotropy restrictions. This model set was clustered using DAMCLUST 55. The biggest cluster consisting of 18 models for the HBXIP‐C7orf59 dimer was averaged and superposition of the final model on the crystal structure of HBXIP‐C7orf59 was done with SUPCOMB 56 after removing the flexible N terminus of C7orf59 (residues 1–15). Images were generated using pymol 1.7 43. Computational jobs were automated with C shell scripts.

### Cell culture, transfection, and coimmunoprecipitation

HEK293‐T cells were grown in Dulbecco's modified Eagle's medium containing 10% FBS at 37 °C with 5% CO_2_. Transient transfections were performed with polyethylenimine 57. Cells were transfected with FLAG‐tagged C7orf59 wild‐type or mutants S67D and S67A. After 48 h of transfection, cells were treated with Forskolin 80 µm or the PKA inhibitor H‐89 (40 µm), both from (Sigma Aldrich, St. Louis, MO, USA). Cells were lysed in lysis buffer [150 mm NaCl, 30 mm Tris, 3 mm EDTA and 0.3% (v/v) IGEPAL CA‐630 pH 8.0] supplemented with phosphatase inhibitor (10 mm beta glycerophosphate and 20 mm NaF) and incubated with FLAG‐agarose affinity beads (Sigma Aldrich) for 3–4 h at 4 °C. The beads were washed four times with lysis buffer and bound proteins were eluted in Laemmli buffer. The samples were analyzed by western blot with antibodies anti‐FLAG (Sigma), anti‐p18/LAMTOR1, and anti‐RagA (Cell Signaling Technology, Danvers, MA, USA).

### *In vitro* phosphorylation

HBXIP‐C7orf59 dimer (WT and S67D), GST‐p18 ΔN + HBXIP‐C7orf59 trimer, and MP1‐p14 dimer were purified by affinity chromatography with Talon beads. GST (negative control) was purified with affinity chromatography using Glutathione‐Sepharose beads. The samples (3.5 µg) were phosphorylated in a 20 µL reaction containing 1250 units of murine PKA (New England Biolabs, Ipswich, MA, USA) and 500 µCi·mmol^−1^ [γ‐32P] ATP in buffer 50 mm Tris (pH 7.5), 10 mm MgCl_2_, 0.2 mm ATP. The reactions were incubated at 30 °C for 30 min and stopped with the addition of Laemmli sample buffer and incubation at 95 °C for 5 min. The samples were analyzed by SDS/PAGE followed by autoradiography detection.

### Identification of the phosphorylation site

Samples from immunoprecipitation of FLAG‐tagged C7orf59 expressed in HEK293 cells were analyzed by SDS/PAGE followed by MS analysis. Briefly, bands of interest were excised, reduced with 10 mm DTT for 30 min, alkylated with iodoacetamide for 30 min in the dark, and in situ digested. Trypsin was added to the gel pieces (enzyme: substrate ratio ~ 1 : 50), and gel pieces were incubated initially at 4 °C for 30 min and overnight at 37 °C. Peptides were extracted, and acetonitrile was evaporated using a speed vacuum before MS analyses were performed. MS analysis were performed on an Orbitrap Fusion Lumos (Thermo Scientific) equipped with a EasyNanoLC 1200 (Thermo Fisher Scientific). Mobile phase A was 0.1% (v/v) formic acid in water. Mobile phase B was 0.1% (v/v) formic acid in acetonitrile. Peptides were separated running a linear gradient from 2 to 30% mobile phase B on a Acclaim™ PepMap™ 100 C18 LC Columns (Thermo Scientific), 3 µm, 150 mm length analytical column kept at 40 °C with 0.3 μL·min^−1^ flow over 120 min. MS data were analyzed in the Orbitrap in the range 400–2000 *m/z* at resolution of 120 000 (at *m/z* 400). The RF lens was set to 25%. The maximum injection time was set to 50 ms with automatic gain control (AGC) 10^6^. The quadrupole was used for precursor ion isolation with window of 1.5* m/z.* The peptides were acquired using data‐dependent analysis by selection of the ten most intense ions (top 10). Precursor ions were fragmented in high energy collision dissociation by applying stepped normalized collision energy fragmentation (40 ± 5%NCE). MS2 were acquired at 15 000 (AGC 2 × 10^4^, 200 ms max. injection time).

## Conflict of interest

The authors declare no conflict of interest.

## Author contributions

Conceptualization: JHCS, DMS, and NR; methodology: JHCS, NR, DMS, LBP, KS, RA, AFZN, FCG, and TBL; investigation: NR, TBL, GLR, ALSS, and JHCS; writing—original draft: JHCS and NR; writing—review and editing: JHCS and NR. with input from other authors; formal analysis: RA, AFZN, TBL, FCG, and GFM; funding acquisition: JHCS, FCG, and RA; visualization: JHCS, NR, and TBL; resources: JHCS, DMS, LBP, KS, and FCG; project administration and supervision: JHCS.

### Data accessibility

PDB: 5VOK.

## Supporting information


**Fig. S1**. Packing of the dimers in the crystal lattice stabilizes the flexible N‐terminal region of C7orf59.
**Fig. S2**. Uncropped versions of Western Blots.
**Fig. S3**. Sequence coverage of HBXIP and C7orf59 in the HDX‐MS experiment.
**Fig. S4**. Identification of p18 degradation products after spontaneous proteolysis and dissociation of the GST‐p18ΔN/HBXIP‐C7orf59 complex.
**Fig. S5**. Small angle X‐ray scattering analysis of the HBXIP‐C7orf59 dimer bound to p18‐ derived peptide.
**Fig. S6**. Size exclusion chromatography showing assembly of pentameric Ragulator.
**Table S1**. Mutagenic primers.
