# Supplementary material for: C7orf59/LAMTOR4 phosphorylation and structural flexibility modulate Ragulator assembly
Source: FEBS Open Bio. 2019 Jul 28;9(9):1589–602. doi: 10.1002/2211-5463.12700 (PMC6722880; doi:10.1002/2211-5463.12700)
Supplement: Supplementary file 1 — Fig. S1. Packing of the dimers in the crystal lattice stabilizes the flexible N‐terminal region of C7orf59. Fig. S2. Uncropped versions of Western Blots. Fig. S3. Sequence coverage of HBXIP and C7orf59 in the HDX‐MS experiment. Fig. S4. Identification of p18 degradation products after spontaneous proteolysis and dissociation of the GST‐p18ΔN/HBXIP‐C7orf59 complex. Fig. S5. Small angle X‐ray scattering analysis of the HBXIP‐C7orf59 dimer bound to p18‐ derived peptide. Fig. S6. Size exclusion chromatography showing assembly of pentameric Ragulator. Table S1. Mutagenic primers. [file FEB4-9-1589-s001.pdf]

Supporting Information

C7orf59/Lamtor4 phosphorylation and structural flexibility modulate Ragulator assembly

Nadia Rasheed[1, 2], Tatiani B. Lima[1, 3], Gustavo F. Mercaldi, Andrey F. Z. Nascimento[4], Ana L. S. Silva[1], Germanna L. Righetto[1], Liron Bar-Peled[5-9], Kuang Shen[5-9], David M. Sabatini[5-9] Fabio C. Gozzo[3], Ricardo Aparicio[3], Juliana H. C. Smetana[1]

Supporting Table 1. Mutagenic primers.

Mutation	*Protein*	*Primer Sequence (5' – 3')*	*Type*
K31	p18	CTCCATTGAGAGCTTAGGTAGGGGGGCTGC	Forward
K31	p18	GCAGCCCCCCTACCTAAGCTCTCAATGGAG	Reverse
G35	p18	GGGCTCGGCTCAATTGAGAGCTTTGGTAGGG	Forward
G35	p18	CCCTACCAAAGCTCTCAATTGAGCCGAGCCC	Reverse
K60	p18	ATGTTGCTGGCTGTCTAGGCAAGGATGGAAGAG	Forward
K60	p18	CTCTTCCATCCTTGCCTAGACAGCCAGCAACAT	Reverse
Q74	p18	CTCCATGCCCTATGAGTCTGCAGCAGACACATC	Forward
Q74	p18	GATGTGTCTGCTGCAGACTCATAGGGCATGGAG	Reverse
E80	p18	CGGTCCATGTACTAATGCTGCTCCATGCCC	Forward
E80	p18	GGGCATGGAGCAGCATTAGTACATGGACCG	Reverse
K103	p18	CGGTGGCAGCTTCTACCAATGGGTCAGGC	Forward
K103	p18	GCCTGACCCATTGGTAGAAGCTGCCACCG	Reverse
L108	p18	GCTGGTAAGAGACGGCTACGGTGGCAGCTTCTTC	Forward
L108	p18	GAAGAAGCTGCCACCGTAGCCGTCTCTTACCAGC	Reverse
Q130	p18	TCCTGGAGACCTGCTACAAATCAGAGAACGGG	Forward
Q130	p18	CCCGTTCTCTGATTTGTAGCAGGTCTCCAGGA	Reverse
D149	p18	AACCAGCTCCTCTTTTGCCTACACACGGATCTGAGAAAG	Forward
D149	p18	CTTTCTCAGATCCGTGTGTAGGCAAAAGAGGAGCTGGT T	Reverse
E34A/N35A	C7orf59	GGCTGCCTGCTCATCAGCCGCCAGGTCCCCAGATGA	Forward
E34A/N35A	C7orf59	TCATCTGGGGACCTGGCGGCTGATGAGCAGGCAGCC	Reverse
D36A/E37A	C7orf59	CACTGGCTGCCTGCGCAGCATTCTCCAGGTCC	Forward
D36A/E37A	C7orf59	GGACCTGGAGAATGCTGCGCAGGCAGCCAGTG	Reverse

S67D	C7orf59	GTTCTCCAAAGACCACATCCAGGCGCTTGAAGGGCA	Forward
S67D	C7orf59	TGCCCTTCAAGCGCCTGGATGTGGTCTTTGGAGAAC	Reverse
S67A	C7orf59	CTCCAAAGACCACAGCCAGGCGCTTGAAGGG	Forward
S67A	C7orf59	CCCTTCAAGCGCCTGGCTGTGGTCTTTGGAG	Reverse
C7orf59ΔN	C7orf59	CTCGGCTACCTGGTACTGAGTGAAGGT	Forward
C7orf59ΔN	C7orf59	GGGCCCCTGGAACAGAACTTCCAGGCT	Reverse

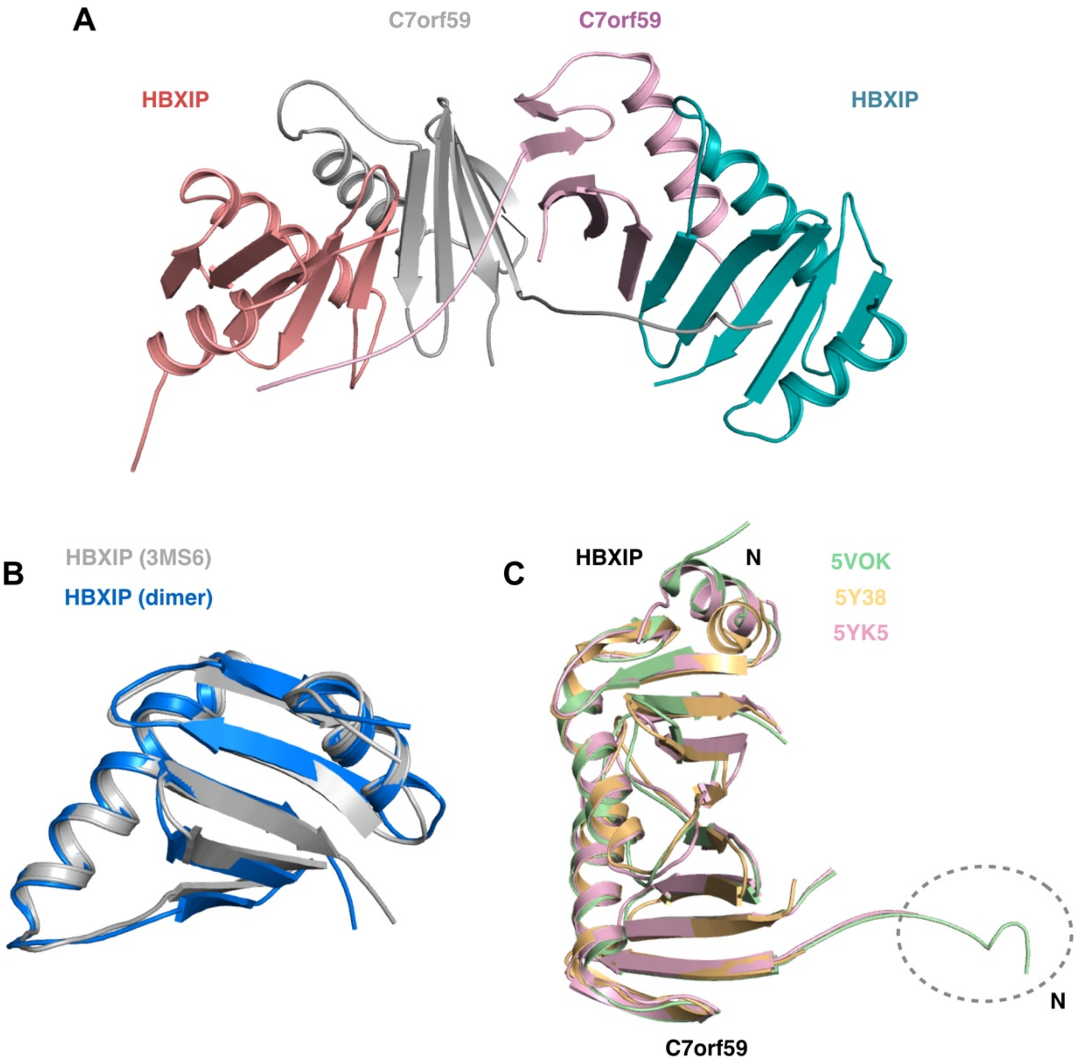

Figure S1. Packing of the dimers in the crystal lattice stabilizes the flexible N-terminal region of C7orf59. Two of the four dimers present in the asymmetric unit are shown. **B:** Superposition of the HBXIP structure reported previously (PDB:3MS6, in grey) and the HBXIP structure from this study in the complex with C7orf59 (blue) with rmsd = 0.622, indicating that it does not undergo major conformational changes upon binding to C7orf59. **C:** Superposition of the crystal structures of HBXIP-C7orf59 dimer from this study (5VOK), Zhang et al (5Y38) and Mu et al (5YK5). The dotted line indicates residues 1–8 of C7orf59 which are visible in the electron density of 5VOK. "N" indicates the N-terminal extremity of each monomer.

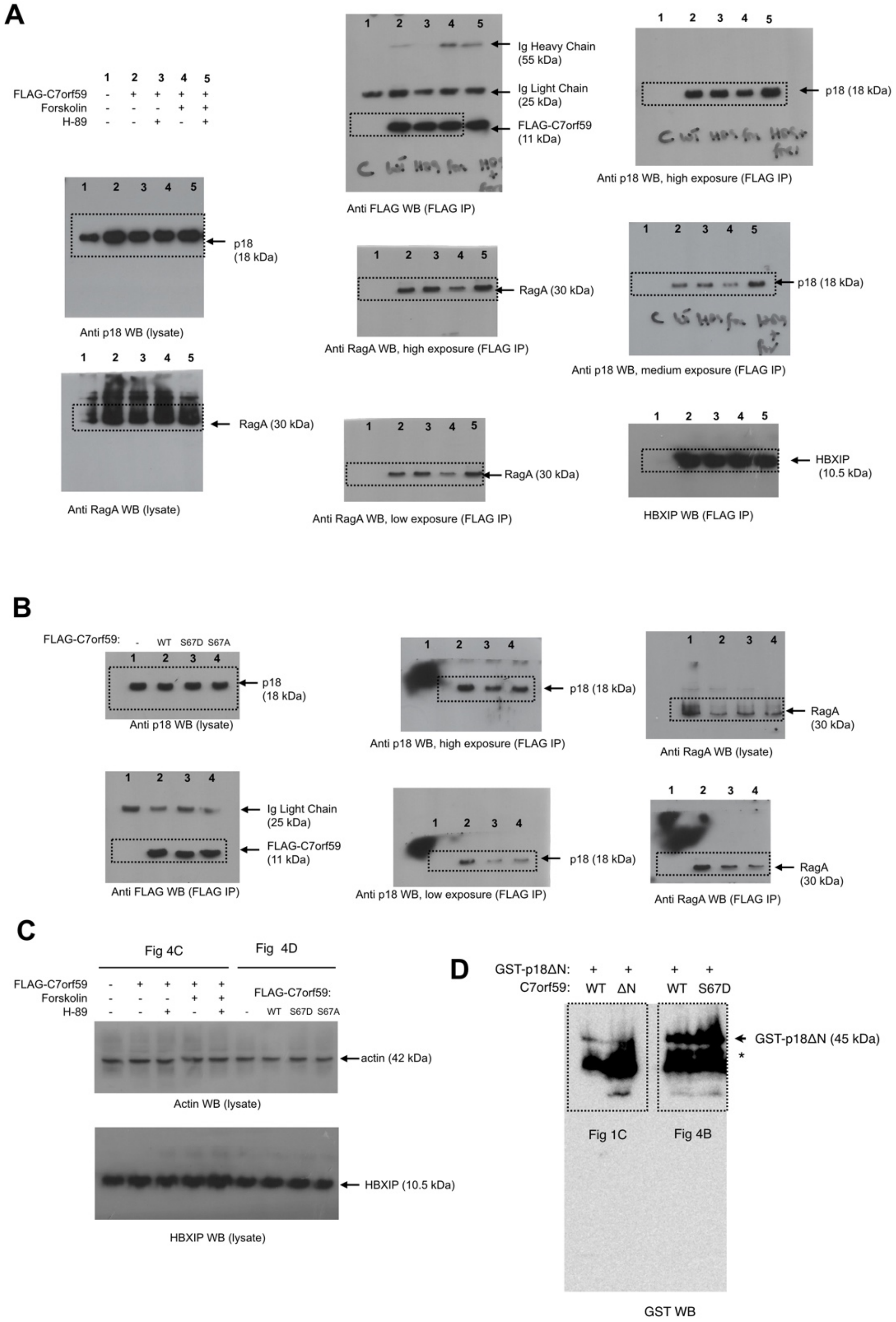

Figure S2. Uncropped versions of Western Blots shown in Fig. 4C **(A)**, 4D **(B)**, 4 C/D **(C)** and Fig. 1C/4B **(D)**.

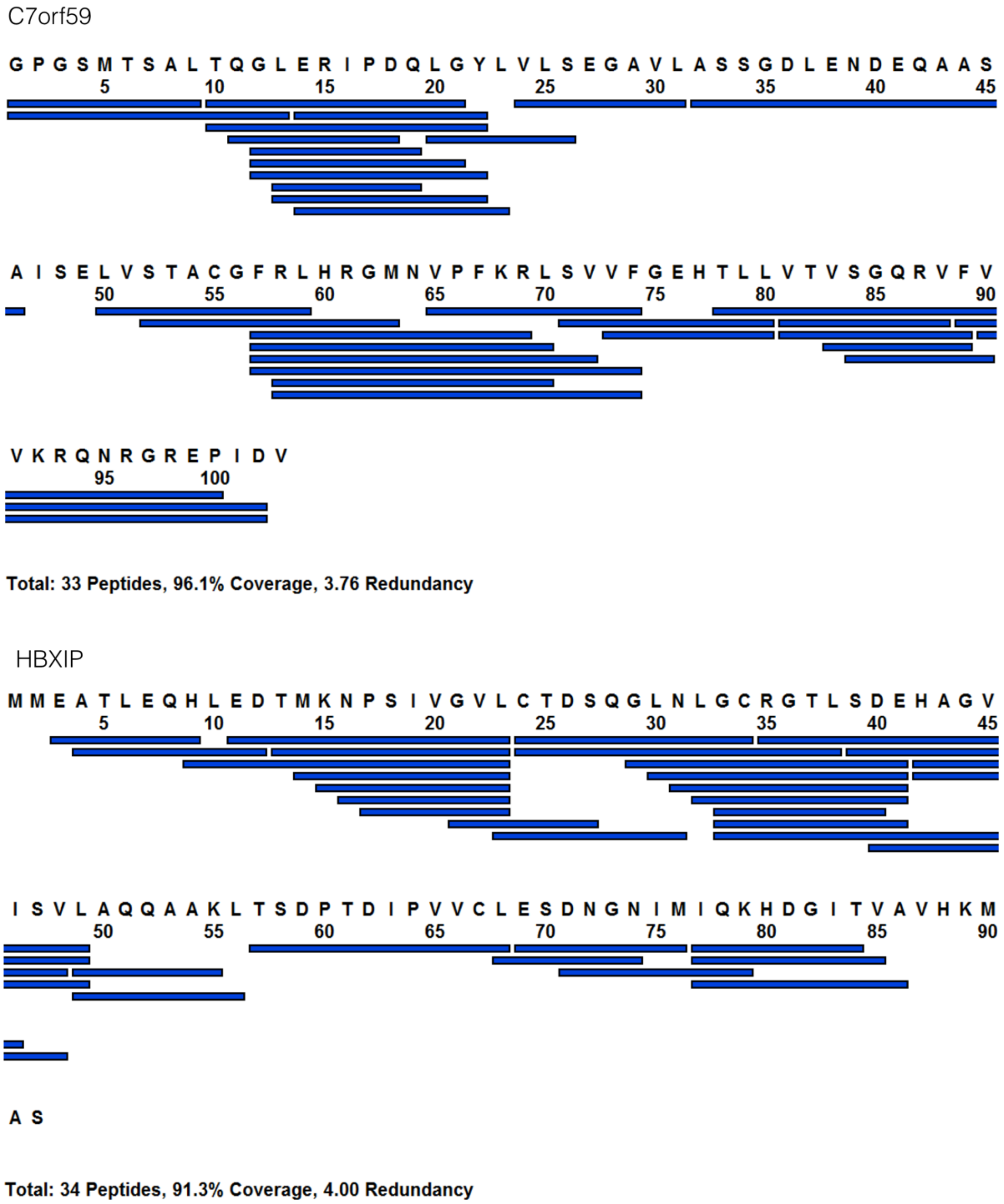

Figure S3. Sequence coverage of HBXIP and C7orf59 in the HDX-MS experiment.

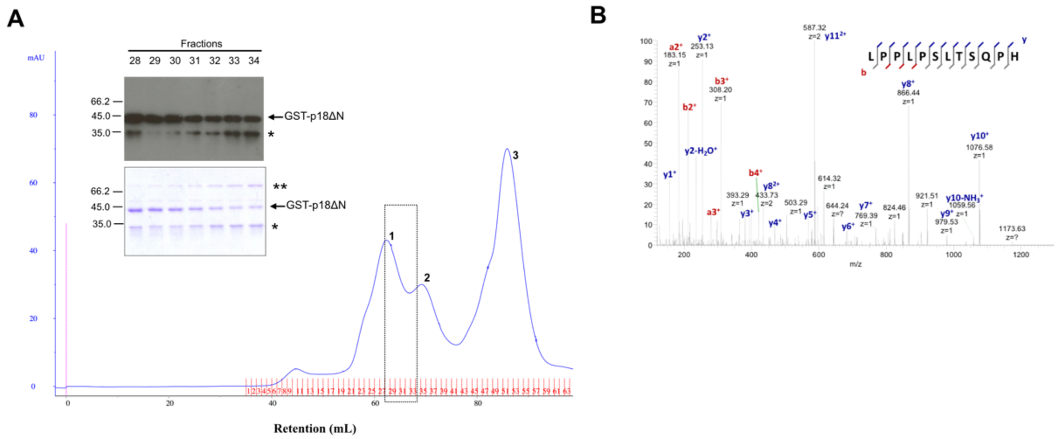

Figure S4. Identification of p18 degradation products after spontaneous proteolysis and dissociation of the GST-p18ΔN/HBXIP-C7orf59 complex. **A:** Size exclusion elution profile of the GST-p18ΔN + HBXIP-C7orf59 complex on a Superdex 200 16/60 column and identification of the ˜35 kD band, identified by an asterisk, as GST-positive. The inset shows GST Western blot (upper panel) and Coomassie-stained SDS-PAGE analysis of selected fractions highlighted by the rectangle. Another unidentified band migrating approximately at 66 kD, marked with a double asterisk, was not recognized by the GST antibody and probably corresponds to a contaminant from *E. coli*. **B:** Mass spectrometry analysis of one of the HBXIP-C7orf59-bound nontryptic peptides (${}_{105}$LPPLPSLTSQPH${}_{116}$) detected from peak 3.

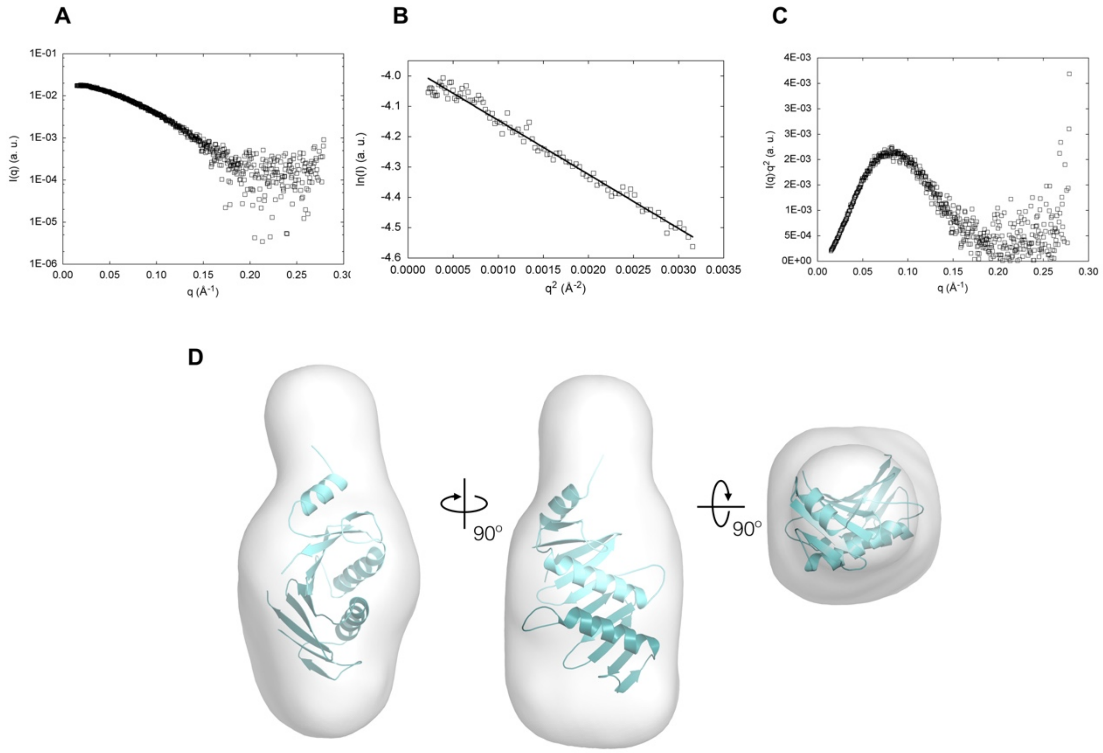

Figure S5. Small angle X-ray scattering analysis of the HBXIP-C7orf59 dimer bound to p18-derived peptide, obtained from spontaneous dissociation of the HBXIP-C7orf59/GST-p18ΔN trimer. **A:** Scattering curve **B:** Guinier region. **C:** Krakty plot. **D:** SAXS envelope fitted to the HBXIP-C7orf59 dimer. The flexible N-terminal residues of C7orf59 were removed for this analysis. HBXIP is in light cyan and C7orf59 is in dark cyan.

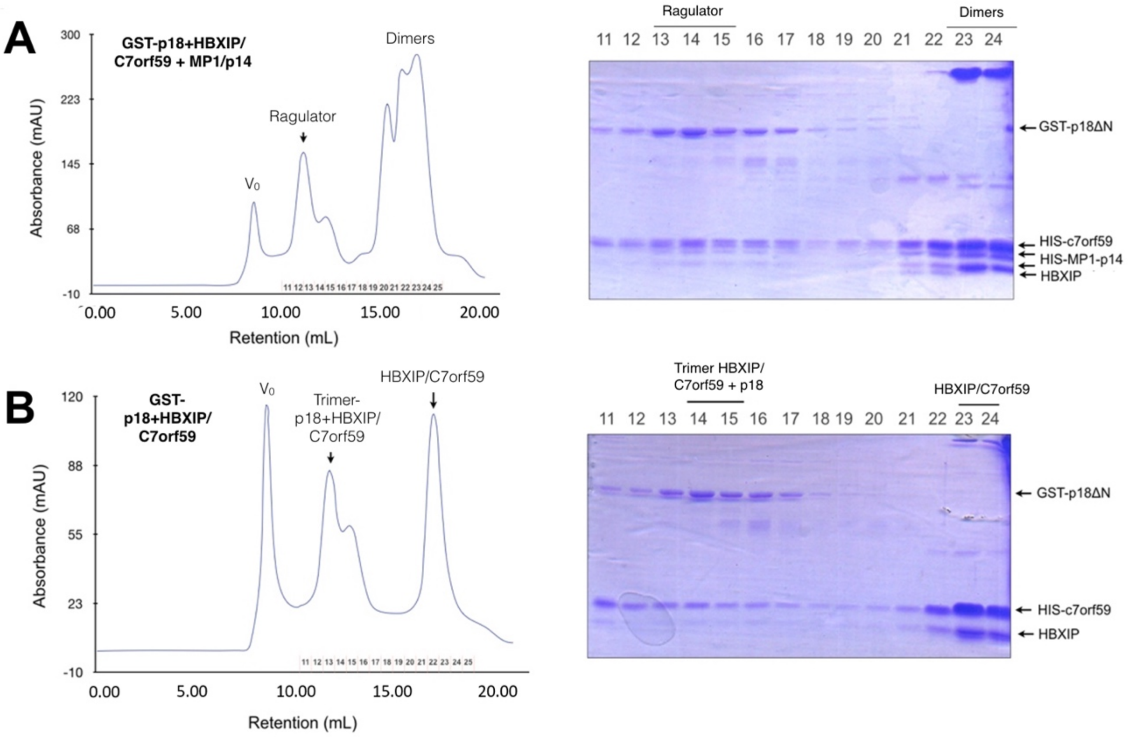

Figure S6. A: Size exclusion chromatography showing assembly of pentameric Ragulator from recombinant, affinity-purified dimer and trimer. Elution profile at 280 nm (left panel) and 16% SDS-PAGE analysis of the fractions eluted from Superdex 200 16/10 size exclusion column (right panel), starting from the affinity-purified MP1-p14 dimer and HBXIP-C7orf59/GST-p18ΔN trimer. **B**: Control run of size exclusion chromatography of HBXIP-C7orf59/GST-p18ΔN trimer under the same conditions, using the same trimer sample that was used for the assembly of pentamer showed above. Left panel: UV (280 nm) elution profile. Right panel: SDS-PAGE analysis of the trimer and dimer fractions.
